# “Long COVID-19” of Researchers: What to Do Next?

**DOI:** 10.1097/HS9.0000000000000673

**Published:** 2021-12-22

**Authors:** Eleni Gavriilaki, Alba Maiques-Diaz

**Affiliations:** 1 Hematology Department-BMT Unit, G Papanicolaou Hospital, Thessaloniki, Greece; 2 Biomedical Epigenomics Group, IDIBAPS/Clinic Hospital, Barcelona, Spain

Coronavirus disease 2019 (COVID-19) pandemic has dramatically changed every aspect of human activity worldwide. Since early 2020, nations worldwide have been on prolonged lockdown periods lasting for several months. These resulted not only in obstruction of biomedical activities and clinical research but also of routine clinical care, along with a dramatic socioeconomic impact. Given that early career researchers (ECRs) are particularly vulnerable in this setting, we launched an initiative to understand the needs of academic/translational researchers (hereinafter referred as lab scientists [LSs]) and clinicians/physician-scientists (hereinafter referred as clinical scientists [CSs]). The effect of this first period was reflected by 2 online surveys led by the Young committee of the European Hematology Association (EHA) with participation of European and international researchers and led to a call to action for ECRs.^1^ As shown by our previous data and others,^2,3^ COVID-19 immediate effect on ECRs has been particularly significant from the first months of the pandemic, likely resulting in long-lasting consequences.

Indeed, the consequences of COVID-19 pandemic continue to impact many aspects of everyday activity, even after months of lockdown, despite a plethora of studies trying to understand and treat the disease, and worldwide vaccination strategies with various vaccination platforms. Therefore, we continued our effort to understand current needs of researchers in the hematology field by creating a follow-up online survey. Since EHA is a comprehensive association including both LS and CS, we were able to gather information and needs of both groups. An invitation was sent to EHA members, responders of the initial surveys and shared through EHA official social media channels.

Our follow-up survey collected data from 70 researchers, 72% of them were based in Europe (ie, Greece, Italy, Germany, Portugal, Romania, Turkey, United Kingdom), and 28% were participants from overseas (ie, Australia, India, United States, Argentina, Brazil). We had good representation of LS (25%) and CS (75%), with most of the responders working on malignant hematology (80%) and a minority in benign hematology (20%). Age shows an even distribution with 40% of them being between 30 and 40 years old, 25% between 40 and 50 years old, and 37% over 50’s (Figure 1A). We decided to analyze the data based on the career stage of responders, which we estimated based on their management responsibilities and not their age. We believe this is a more appropriate representation, as age constraints may vary between countries, and it is difficult to define what an ECR is only based on age. According to this, most participants (47/70) were established researchers that had full managing responsibilities, likely corresponding to principal investigators or group leaders. A quarter of responders (16/70) had partial management responsibilities, corresponding to postdoctoral researchers or similar, and very few (8/70) indicated they had no management responsibilities, meaning they may be students or similar. There were slightly more female (56%) than male (44%) responders.

**Figure 1.**
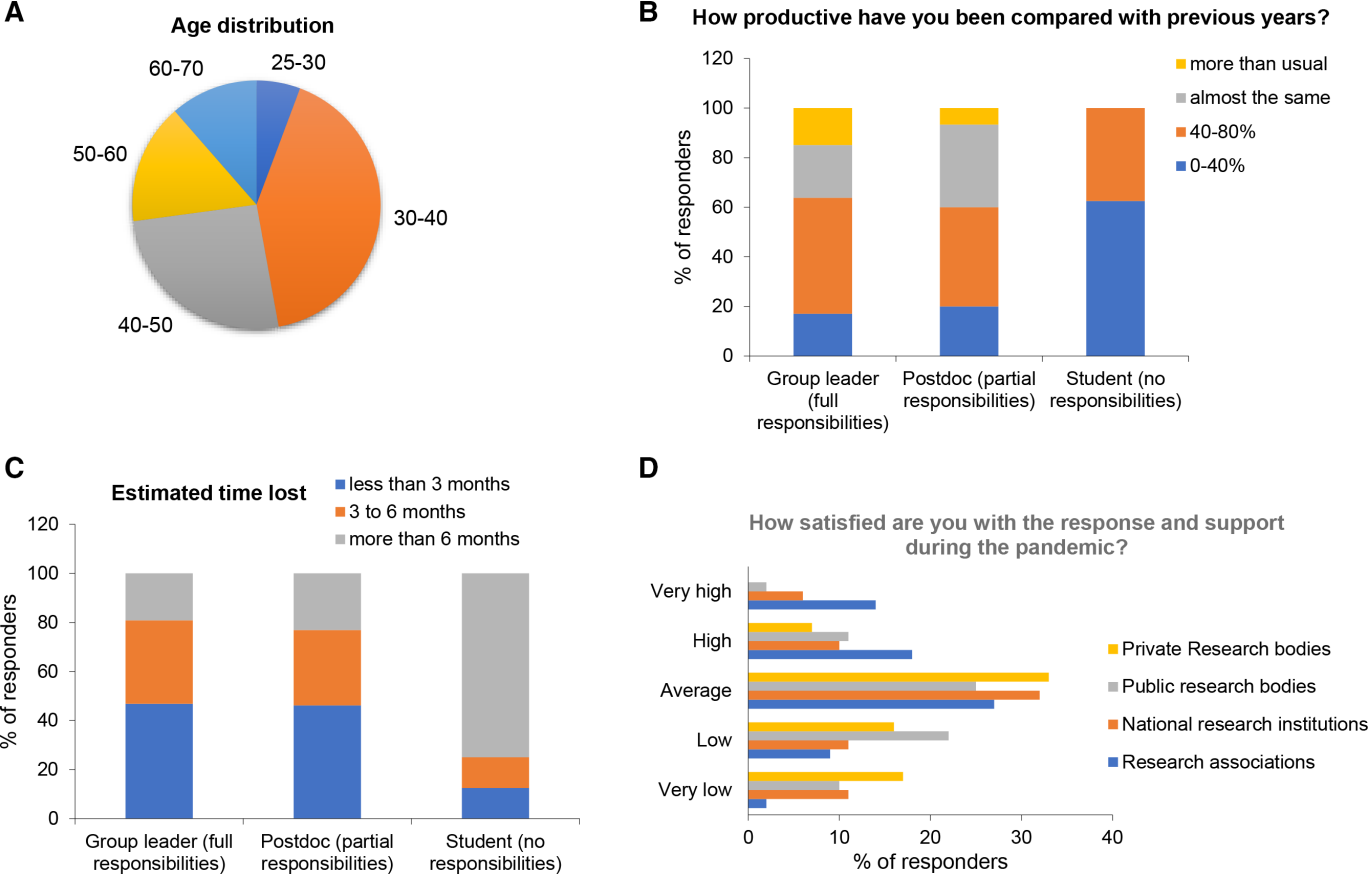
**Summary of the characteristics of the responders to the follow-up survey launched by Young EHA committee.** (A), Age distribution of the participants. (B and C), Bar graph summarizes the reported impact in (B) productivity (ie, answer to the question “Overall, thinking about the last 12 months I would estimate that the total amount of time lost related with my work would be”) and (C) overall time lost (ie, answer to the question: “How productive you have been during the last 12 months compared to previous years?”) divided in 3 groups corresponding to their career stage (assessed by their managing responsibilities). (D), Bar graph summarizing the number of participants according to the rate of satisfaction they reported to the questions “How satisfied are you with the response and support during the pandemic from (1) private research bodies, (2) public research bodies, (3) national research institutions, and (4) research associations?” EHA = European Hematology Association; PIs = full responsibility/principal investigator or similar; Post-doc = partial responsibility/postdoctoral researcher or similar; Student = No responsibility/PhD student, medical student, or similar.

As in our previous surveys, we first focused our attention on the time lost related to the participants’ work during the pandemic (time loss) and how productivity was affected. We asked responders to consider the last 12 months, meaning that the time covered by the survey was, approximately, from June 2020 to June 2021. During that time frame, half of the participants experienced a lockdown of less than 6 months (52%), a third between 6 and 12 months (35%), and a small but still significant minority, of more than 12 months (13%). However, lockdown is only one measure of the impact on their work; thus, we also asked them about their productivity and their overall time loss. Although these are both quite personal and may be biased on individualized perceptions, when combined, they may reflect the real impact of the pandemic.

Most responders documented a lower productivity compared to the pre-COVID 19 situation. However, 11% reported to be “more productive” and 20% to be “equally productive.” Similarly, the “total amount of time lost related with my work” was estimated at 0–3 months by many participants (41%, 29/70), while less than 30% of the responders indicated a time loss of 3–6 months (28.5%, 20/70) or even longer (6–9 months, 18.6%, 13/70), respectively. We did not find significant differences in the reported impact on productivity/time lost between age or gender groups. However, principal investigator´s work (ie, people with managing responsibilities) was overall less impacted by time loss or lower productivity, while people that did not have responsibilities indicated higher amount of time loss and less productivity due to the pandemic (Figure 1B and C). The main reasons attributed to productivity loss were the necessity to stop the experiments during lockdown, as well as stress and anxiety (26/70 responders for both). Interestingly, among 37 responders in need of childcare support, only 10 had that support through their family. Furthermore, the pandemic impacted the recruitment of new members for research endeavors, which was indicated by 48% of participants. More importantly, support by funding bodies was reported only by 10% of responders, slightly increasing in support of medical associations (approx. 20%). In line with these statements, responders showed a mean satisfaction of 3.47 (SD: 1.05; scale 1–5, with 5 being very satisfied) with the response of research associations, as depicted in Figure 1D. Contrary to that, the satisfaction with policy bodies and stakeholders was clearly less pronounced (2.61, SD: 1.01). Lastly, in an effort to call to action, our participants were asked for recommendations ensuring high-quality research and funding in the near future. Table 1 summarizes some of the biggest concerns reported for the post-COVID-19 era, as well as some useful resources suggested by the participants.

**Table 1.** Summary of Biggest Concerns Reported for the Post-COVID-19 Era, As Well As Some Useful Resources Suggested by the Participants.

Reported Concerns	Suggested Useful Resources
Another lockdown/new COVID-19 variants/effectiveness of vaccination	Having casual chats with peers rather than formal “support” meetings
Limited opportunities for young researchers and principal investigators	Resources such as the EHA and Medscape; also WHO/PAHO
Securing funding/grants	International organizations
Socioeconomic impact	Reduce bureaucracy
Childcare/gender equity	Virtual seminar series

COVID-19 = coronavirus disease 2019; EHA = European Hematology Association; PAHO = Pan American Health Organization; WHO = World Health Organization.

Regarding vaccinations, the vaccination rate was high among responders (55/70) and this involved also other researchers working with them (52/70). Interestingly, unvaccinated responders were mostly LS (6/11) and came from Portugal, Russian Federation, Romania, Bulgaria, Italy, Australia, United Kingdom, India, Spain, Germany, and Azerbaijan. Only a minority of responders were involved with clinical care of COVID-19 patients or hematological patients with COVID-19 (19/70 and 18/70, respectively).

Overall, our follow-up survey has confirmed that COVID-19 continues to have a significant impact on researches, in particular those at early career stages such as at postdoctoral, doctoral, or student levels. These researchers suffered from detrimental and continuous loss of productivity and work-life balance. Despite several concerns and calls for actions raised worldwide on supporting ECRs,^4–8^ our initiative showed dissatisfaction of researchers with the actions taken by organizations and policy makers regarding biomedical research. To gather information on what type of actions researchers would find most useful, we asked them to rate the top recommendations from a fixed list. As expected, the great majority answered that providing additional grant opportunities and reducing bureaucracy will alleviate their current situation. Many responders also suggested other actions related to funding, such as the reduction of the requirements for preliminary data in grant applications or offering no-cost extensions of grants, and some of them consider reasonable to take into account pre-prints in grant applications. When asked about research in general, many responders considered good initiatives to provide better tools for education of young researchers and to change the evaluation criteria of research. Although these topics are quite broad and may need further debate within the research community, we believe are good starting points to be considered by all stakeholders involved in biomedical research. Young researchers deserve creative solutions to overcome the “long COVID-19” effect research is going through. We appeal to ECRs, research associations, funding bodies, and public agencies to take into account these data for future actions and initiatives.

In conclusion, despite several concerns and calls for actions raised worldwide on supporting researchers,^4–8^ our initiative showed dissatisfaction of researchers with the actions taken by policy makers regarding biomedical research. To overcome this “long COVID-19” effect on researchers, we call all stakeholders to take action.

## ACKNOWLEDGMENTS

We would like to thank everyone that shared their experiences in the surveys, Deepa Maas-Sundararaman for her support and advice in the study, and Dr Benedikt Pelzer for his contribution.

## DISCLOSURES

EG is supported by the ASH Global Research Award. AMD is supported by the Beatriu de Pinós Programme of the Government of Catalonia (2018-BP-00231).
